# Cryptococcus neoformans Coinfection Dampens the TNF-α Response in HIV-1-Infected Human THP-1 Macrophages

**DOI:** 10.1128/mSphere.00213-21

**Published:** 2021-03-24

**Authors:** Murat C. Kalem, Monica S. Humby, Elizabeth A. Wohlfert, Amy Jacobs, John C. Panepinto

**Affiliations:** a Department of Microbiology and Immunology, Witebsky Center for Microbial Pathogenesis and Immunology, Jacobs School of Medicine and Biomedical Sciences, University at Buffalo, SUNY, Buffalo, New York, USA; University of Georgia

**Keywords:** *Cryptococcus neoformans*, HIV-1, NF-κB, TNF-α, coinfection

## Abstract

Cryptococcus neoformans is a devastating opportunistic fungal pathogen. It mostly impacts people in an immunocompromised state, such as people living with HIV/AIDS and following organ transplantation. Macrophages, in addition to being a major cellular reservoir of HIV-1, represent a unique niche in which both C. neoformans and HIV-1 can coinhabit in the course of natural infection. Here, we report the observation that HIV-1 infection of THP-1 macrophages increases the rate at which they phagocytose C. neoformans cells. We investigated the tumor necrosis factor alpha (TNF-α) signaling and nuclear factor kappa B (NF-κB) activation in human monocyte-derived macrophages infected with HIV-1 alone, as well as those coinfected with HIV-1 and C. neoformans. Our findings showed that while HIV-1 infection alone upregulates TNF-α production and activates NF-κB signaling, C. neoformans coinfection drastically and rapidly dampens this proinflammatory response. These data suggest an antagonism between two important human pathogens during coinfection of macrophages.

**IMPORTANCE** Fungal infections are one of the leading causes of death for people who live with HIV/AIDS. Even though these pathogens are independently well studied, it is still enigmatic how coinfection with HIV-1 and C. neoformans alters gene expression and cellular processes, especially in clinically relevant cell types. Understanding the interplay between these two pathogens is especially critical because C. neoformans mortality largely depends on the host’s immunocompromised state during viral infection. Studying this coinfection is challenging since HIV-1 only infects human cells, and the modified murine HIV-1 virus does not reproduce the clinical landmarks of HIV-1 infection or AIDS in mice. Our observations shed light on how these two pathogens trigger opposing trends in TNF-α and NF-κB signaling in human monocyte-derived macrophages.

## OBSERVATION

The fungal pathogen Cryptococcus neoformans causes 15% of HIV-related deaths worldwide, and yet the effects of coinfection with HIV-1 and C. neoformans on each infection’s pathogenesis are largely unstudied (1). Macrophages are pivotal effectors in the pathogenesis of both HIV-1 and C. neoformans, representing an important reservoir for HIV-1 latency (reviewed in reference 2) and potentially for cryptococcal latency as well (3). Proinflammatory immune responses in immunocompetent individuals promote an M1 macrophage phenotype that can clear C. neoformans and relegate infection to the lung (4). In the absence of T-cell help, C. neoformans can skew macrophage activation toward an M2, nonprotective phenotype through the effects of the immunomodulatory capsule and production of prostaglandin E_2_, among other effectors (reviewed in references [Bibr B5][Bibr B6][Bibr B8]). Macrophages are a vehicle for disseminating HIV-1 and C. neoformans to the central nervous system (9, 10), where HIV-1 causes neurodegeneration and C. neoformans causes a meningoencephalitis that is uniformly fatal without “gold standard” antifungal therapy. Even though we know how single infections with C. neoformans or HIV-1 impact macrophages, it is unclear how the coinfection is established and how it modulates macrophage function (11, 12).

Given that (i) HIV-1 infection of macrophages induces proinflammatory tumor necrosis factor alpha (TNF-α) production (13, 14) and (ii) C. neoformans exhibits anti-inflammatory effects (reviewed in reference 5), we set out to investigate the impact of coinfection on the inflammatory activation of macrophages. C. neoformans induces phagosomal membrane permeabilization in macrophages, leading to apoptosis (15). In contrast, gp120 of HIV-1 can exhibit antiapoptotic effects through the upregulation of MCP-1 (16). Thus, HIV-1-infected macrophages interacting with C. neoformans will experience both anti- and proapoptotic signals.

One of the major factors that control TNF-α production is the transcription factor nuclear factor kappa B (NF-κB). C. neoformans capsular polysaccharide GXM inhibits the lipopolysaccharide (LPS)-induced translocation of the NF-κB subunit p65 to the nucleus in murine macrophages. Conversely, intracellular C. neoformans allows the LPS-induced p65 translocation (17, 18). NF-κB is constitutively activated in HIV-1-infected monocytes. Nuclear NF-κB binds to the enhancer region of the HIV-1 long terminal repeat (LTR), which carries two adjacent NF-κB binding sites. NF-κB activation allows high levels of HIV-1 gene expression and favors viral replication (19, 20). NF-κB activity responds to C. neoformans and HIV-1 infections differently, and NF-κB activation during coinfection of humans is unexplored.

Here, we report an observation that HIV-1-infected THP-1 macrophages exhibit increased phagocytosis of C. neoformans, NF-κB activation, and TNF-α gene expression, consistent with proinflammatory activation. Once exposed to C. neoformans, however, the proinflammatory markers are rapidly repressed in a manner dependent on the whole, live C. neoformans. These results suggest that C. neoformans dominates the macrophage response during coinfection, antagonizing the proinflammatory activation of HIV-1 infection.

### HIV-1-infected human THP-1 macrophages have an increased rate of C. neoformans phagocytosis.

**FIG 1:**
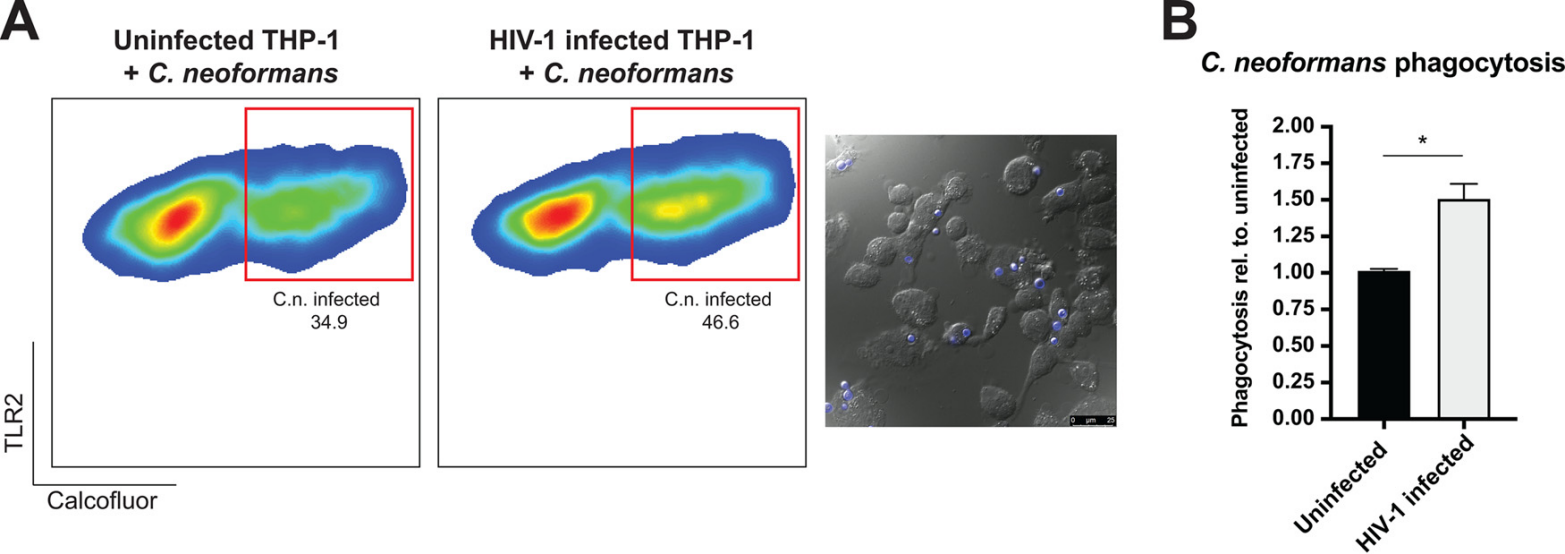
HIV-1-infected human THP-1 macrophages have an increased rate of C. neoformans phagocytosis. (A) Flow cytometry analysis of the C. neoformans-infected THP-1 macrophages. C. neoformans-infected population is indicated in the red boxes. Representative image showing the phagocytosis of C. neoformans (blue, calcofluor white) by THP-1 macrophages. (B) Quantification of the phagocytosis rate. The percentages of the population of the C. neoformans-infected macrophages were analyzed and graphed relative to phagocytosis in the uninfected population. The error bar shows the standard error of the mean (SEM). A *t* test with Mann-Whitney test was performed to show significance.

Recognition and phagocytosis of C. neoformans by macrophages are necessary host responses to fight and clear the C. neoformans infection in the lungs. The immunocompromised state of the host may alter this interplay. We investigated the phagocytosis of C. neoformans following HIV-1 infection in differentiated THP-1 macrophages (Fig. 1A and B). THP-1 monocytes were differentiated to macrophages and infected with HIV-1. Infected macrophages were coinfected with calcofluor-stained C. neoformans strain H99. Toll-like receptor 2-positive (TLR2^+^) cells were gated to eliminate the free C. neoformans and isolate analysis to the THP-1 macrophages. Flow cytometry analysis revealed that HIV-1-infected THP-1 cells phagocytosed C. neoformans ∼30 to 50% more than uninfected cells. Microscopy images are shown to confirm phagocytosis (Fig. 1A; blue, calcofluor-stained C. neoformans).

It was previously shown, by Seoane and colleagues, that phagocytosis of C. neoformans by human peripheral blood mononuclear cell (PBMC)-derived macrophages did not change following infection with pseudotyped and replication-defective HIV-1 (21). Our study utilizes a fully infectious virus compared to pseudotyped and replication-defective HIV-1 containing the vesicular stomatitis virus envelope protein, which may activate different immune signaling pathways that can explain the observed differences. Multiple rounds of viral infection due to viral replication might activate a more robust signaling through the engagement of CD4, CXCR4, and CCR5 receptors, and the envelope protein gp120 can, itself, signal through Toll-like receptors (22, 23). This might create a macrophage population that is more readily differentiated. Additionally, Seoane and colleagues utilized human serum or 18B7 capsule antibody for opsonization, compared to the rabbit complement opsonization in our study. Complement-mediated phagocytosis acts through complement receptors (CRs), especially CR3, whereas antibody-mediated phagocytosis occurs through Fc receptors (summarized in reference 24). These nuances in experimental designs using model systems may result in differential cellular signaling that may influence outcome and should be considered while comparing results across multiple studies. While THP-1-derived macrophages are proven to be an effective model, it is not without its caveats.

### C. neoformans infection of HIV-1-infected human THP-1 macrophages antagonizes TNF-α expression.

**FIG 2:**
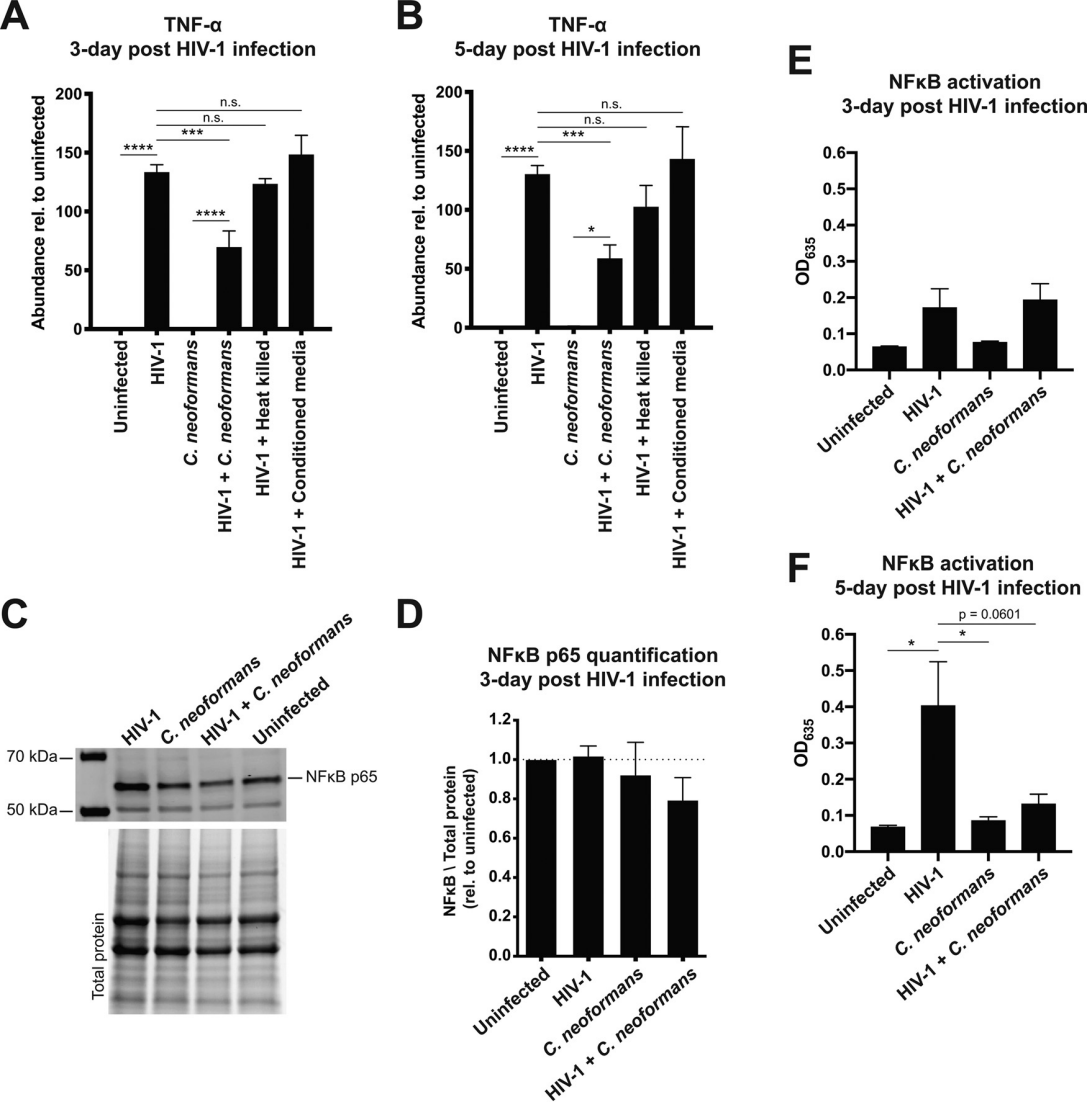
C. neoformans infection of HIV-1-infected human THP-1 macrophages dampens TNF-α levels and NF-κB signaling. (A and B) Infection for (A) 3 or (B) 5 days with HIV-1 upregulated the TNF-α expression, while C. neoformans infection of HIV-1-infected macrophages dampened the TNF-α transcript abundance. TNF-α abundance was determined using RT-qPCR. Heat-killed cells and conditioned medium did not yield a significant change. (C) Immunoblot analysis probing for the NF-κB subunit p65. The bottom panel shows the total protein as a loading control. (D) Quantification of the p65 bands showed no change in the protein levels. p65 was normalized to total protein and graphed relative to uninfected THP-1 macrophages. (E) NF-κB activity at 3 days post-HIV-1 infection. THP-1 Blue cells were infected with indicated pathogens, and cell culture medium was collected. NF-κB activity was detected using a secreted alkaline phosphatase assay. (F) NF-κB activity at 5 days post-HIV-1 infection. The error bar shows the standard error of the mean (SEM). One-way analysis of variance (ANOVA) with Tukey’s multiple-comparison test was performed to show significance.

Infection of macrophages with HIV-1 is reported to induce TNF-α expression. Following HIV-1 infection of THP-1 macrophages, we first hypothesized that TNF-α would be induced upon infection. We found that on days 3 and 5 post-HIV-1 infection, TNF-α expression increased more than 100-fold (Fig. 2A and B). Exposure of HIV-1-infected macrophages to rabbit complement-opsonized C. neoformans for 2 h on days 3 and 5 reduced TNF-α mRNA by half. Rabbit complement alone was not enough to reduce the TNF-α mRNA levels (data not shown.) Exposure of uninfected, age-matched macrophages to C. neoformans resulted in the predicted absence of TNF-α induction. Exposure of HIV-1-infected macrophages to either heat-killed C. neoformans or conditioned RPMI medium had no significant effect on TNF-α expression, suggesting that the effect required live C. neoformans.

### C. neoformans infection of HIV-1-infected human THP-1 macrophages dampens HIV-1-induced NF-κB signaling.

The major regulator of TNF-α expression is the nuclear factor kappa B (NF-κB). NF-κB is a transcription factor with widespread impacts on the regulation of genes involved in innate and adaptive immune responses. To determine if the downregulation of TNF-α gene expression was mediated by a reduction in the activity of NF-κB, we assessed NF-κB activation by two methods. We first determined the levels of p65, an NF-κB subunit, by Western blotting and observed a minor, statistically nonsignificant decrease in p65 levels during coinfection. The levels are unchanged in both the uninfected cells and cells infected with either HIV-1 or C. neoformans (Fig. 2C and D). Even though there are no significant changes in the NF-κB subunit levels, this regulation might be controlled through nuclear translocation rates rather than abundance. We then sought to quantify NF-κB activation by using the THP-1 Blue NF-κB reporter cell line. We collected cell culture media and analyzed the NF-κB activity through a colorimetric assay. On days 3 and 5 post-HIV-1 infection, NF-κB activation was increased in HIV-1-infected cells (Fig. 2E and F). Exposure of these cells to C. neoformans opsonized with rabbit complement for 2 h was sufficient to repress NF-κB activation. This suggests that C. neoformans represses TNF-α gene expression, at least in part, through the regulation of NF-κB activity.

Our finding that C. neoformans dampens the TNF-α immune response suggests a mechanism in which fungal cells remodel intracellular signaling pathways in the immunocompromised host. Fungal infections mostly arise as opportunistic infections, and studies investigating the interplay between HIV-1 and C. neoformans are needed. NF-κB activation and downstream signaling events control many cellular processes, including gene expression, ubiquitin proteasome activation, and apoptosis. NF-κB controls the expression of antiapoptotic genes and is activated by HIV-1 to increase survival. Our observations lay out the groundwork to further investigate immune modulation by C. neoformans during coinfection in human monocyte-derived THP-1 macrophages. Future work will investigate the mechanism by which C. neoformans represses TNF-α expression induced by HIV-1 infection both through regulation of NF-κB activation and potential posttranscriptional regulation of the TNF-α mRNA.

### Cell culture and HIV-1 propagation.

Human monocytic THP-1 cells (ATCC) were grown in RPMI 1640 medium supplemented with 100 U/ml penicillin, 100 μg/ml streptomycin, 10% heat-inactivated fetal bovine serum, and 0.05 mM 2-mercaptoethanol in a 37°C static incubator containing 5% CO_2_. The HIV-1 RF strain (acquired from the NIH AIDS Reagents Program, catalog no. 2803, lot no. 110021) was propagated by coculturing with 293T cells. C. neoformans var. *grubii* strain H99, a fully virulent strain, was a gifted from Peter Williamson (UIC, NIAID), which was derived from H99O (a gift from John Perfect, Duke University). C. neoformans cells were grown overnight at 30°C in yeast-peptone-dextrose (YPD) broth while shaking at 250 rpm.

### THP-1 differentiation and infections.

THP-1 monocytic cells were differentiated to macrophages by treatment with 50 mM phorbol 12-myristate 13-acetate (PMA) for 2 days. At the end of the 2-day differentiation process, nonadhered cells were aspirated, and adhered macrophages were left for 1 day of rest in fresh medium. Macrophages were detached and counted, and each well of a 6-well plate was seeded with 2 × 10^6^ cells. Macrophages were infected with the HIV-1 RF strain for 3 or 5 days at a multiplicity of infection (MOI) of 1. At the end of HIV-1 infection, macrophages were infected with 1 × 10^7^ C. neoformans cells per well. C. neoformans cells were washed three times with phosphate-buffered saline (PBS) and opsonized with 5% rabbit complement (Pel-Freez Biologicals) for 1 h at 37°C while shaking (25–27). Opsonized C. neoformans cells were transferred to wells containing differentiated THP-1 cells and incubated for 2 h at 37°C with 5% CO_2_. C. neoformans cells were heat killed by incubating cells at 65°C for 1 h before infection. Conditioned medium was prepared by incubating C. neoformans cells in RPMI medium for 2 h and sterile filtering the medium.

### Flow cytometry.

HIV-1 and C. neoformans infections were carried out as described above, except C. neoformans cells were stained with 20 μg/ml calcofluor for 10 min at room temperature. Following infections, THP-1 macrophages were washed once with PBS. Then cells were resuspended in 500 μl PBS and transferred to 96-well plates. The 96-well plates were centrifuged at 1,400 rpm for 3 min at 4°C. Cell pellets were resuspended in 50 μl of PBS containing 0.5 μg fluorescein isothiocyanate (FITC)-conjugated TLR2 antibody and 1:500 diluted LIVE/DEAD fixable violet aqua stain. Cells were incubated on ice for 25 min. Then 100 μl of PBS was added to each well, and the plate was centrifuged. Cells were washed once with 150 μl PBS. Cell pellets were fixed in 100 μl 2% paraformaldehyde for 10 min on ice. One hundred microliters PBS was added to each well, and the plate was centrifuged. Cells were washed with PBS once. Cells were then resuspended in 300 μl PBS and transferred to flow cytometry tubes. Flow cytometry was performed using a BD LSR Fortessa cell analyzer, and data were analyzed using FlowJo version 10. C. neoformans-infected THP-1 macrophages were also imaged using a Leica TCS SP8 confocal microscope to confirm phagocytosis of calcofluor-labeled C. neoformans.

### Immunoblotting.

Cells were washed three times with PBS and resuspended in 750 μl PBS containing Roche cOmplete Mini protease inhibitor. Cells were transferred to microcentrifuge tubes and centrifuged at maximum speed for 5 min. The pellet was resuspended in mammalian protein extraction buffer (M-PER; Thermo Fisher) supplemented with protease inhibitors. Lysates were clarified by centrifugation at maximum speed for 15 min, and protein levels were quantified using a Pierce 660 protein quantification kit. Protein samples were run on 4 to 15% TGX stain-free gels (BioRad). The stain-free signal was detected using a Gel Doc XR+ gel documentation system (BioRad). Gels were transferred to nitrocellulose membranes using the TransBlot Turbo system. Membranes were blocked in Odyssey blocking buffer and then incubated overnight at 4°C with 1:1,000-diluted anti-p65 antibody (sc-8008; Santa Cruz Biotechnology) in TBST (Tris-buffered saline with Tween 20) containing 20% blocking buffer. Blots were washed three times and then incubated in 1:10,000-diluted anti-mouse 800CW LiCor secondary antibody for 1 h at room temperature. Blots were imaged using a LiCor Odyssey imaging system.

### RT-qPCR.

RNA was extracted using Trizol, and reverse transcription-quantitative PCR (RT-qPCR) was performed as described previously (28). Briefly, cells were DNase I treated using the Ambion Turbo DNA-free kit (Thermo Fisher). cDNA was synthesized using the Applied Biosystems High-Capacity cDNA Synthesis kit. TNF-a transcript abundance was measured (forward primer 5′-CCTCTCTCTAATCAGCCCTCTG-3′ and reverse primer 5′-GAGGACCTGGGAGTAGATGAG-3′) and normalized to GAPDH (glyceraldehyde-3-phosphate dehydrogenase) abundance (forward primer 5′-TCAAGGCTGAGAACGGGAAG-3′ and reverse primer 5′-TCGCCCCACTTGATTTTGGA-3′).

### NF-κB activity measurement.

THP-1 Blue cells were differentiated and infected as described above. Five hundred microliters of the culture medium was transferred to microcentrifuge tubes and stored at −80°C. NF-κB activity was measured using QuantiBlue secreted alkaline phosphatase detection medium (InvivoGen).
